# How good is the macaque monkey model of the human brain?

**DOI:** 10.1016/j.conb.2009.01.002

**Published:** 2009-02

**Authors:** Richard Passingham

**Affiliations:** Department of Experimental Psychology, University of Oxford University, South Parks Road, Oxford OX1 3UD, United Kingdom

## Abstract

Macaque monkeys are widely used in order to understand the mechanisms of the human brain. But humans have capacities not found in monkeys, and their brains differ in important ways, for example in the proportions of different regions and in microstructure. However, this does not mean that we must abandon the monkey model, only that wherever possible, we should test whether generalizations can be made. One strategy is to use fMRI to visualize activations in humans, and compare these with activations in monkeys. Where the results are the same, we can then use information from single unit recording in those areas to suggest the mechanisms by which those areas perform their functions in the human brain.

## Introduction

Monkeys have been used for studies of the neural mechanisms of cognition for over 70 years [1]. Most of this work has been carried out on macaque monkeys, though there are also studies on marmosets, and a few studies on squirrel monkeys and cebus monkeys. There have been no such studies on great apes such as the chimpanzee or the lesser apes, the gibbons. The assumption has been that studies on monkeys will help us to understand the human brain. There could be two challenges.

The first accepts that these studies could be helpful but argues that they are no longer needed. The claim is that fMRI, MEG, TMS and DTI can now tell us everything that we need to know about the human brain for the purposes of cognitive neuroscience. However, this objection fails to distinguish between methods that record from or disrupt whole populations of cells and methods that record from cells one at once or in small populations. The spatial resolution of imaging methods is adequate if one is interested in the functions of an area. But if one is interested in *mechanism*, that is in how the area does what it does, there is no alternative to using methods with a much finer spatial resolution. The reason is that one needs to know how the different cells interconnect within a module [2], and how the differential coding of each cell within a module contributes to the population signal [3]. It is true that in fMRI multivariate techniques can be used to compare the pattern of activity across voxels in different tasks [4], but so far they have not significantly advanced our knowledge of the detailed mechanisms by which behaviour is coded. Though some progress has been made with receptive field models and plots of the tuning curves for individual voxels [5,6], voxels as typically measured at the moment contain millions of neurones [7]. The scale allows fine mapping, but it is much too coarse if we are to understand the underlying mechanisms.

The objection also assumes that DTI will be able to provide the detail on anatomical wiring that is provided by the use of tracers in the macaque brain. The reason why we need detailed wiring diagrams is that the functions performed by an area are determined both by the pattern of extrinsic inputs and outputs of that area [8^•^] and also by intrinsic wiring of that area [9]. DTI can certainly provide information on the major pathways in the human brain, but it is unlikely that it will be able to achieve the level of detail currently available from tracers, We now have 36 994 connection details on the connections of the cerebral cortex in the macaque monkey brain (www.mon-kunden.de/cocomac). And although specialized coils can be used to enable diffusion imaging to visualize the termination of thalamo-cortical inputs within the cortical layers [10], it is a long way from being able to visualize the details of intra-cortical wiring.

The second challenge is more serious. This is that the lines leading to modern monkeys and humans have been separated for 25 million years [11]. Thus, one would expect to find significant differences between the brains of monkeys and humans [12^••^]. Furthermore, there are very marked behavioural differences [13,14^•^,15], and these must depend partly on differences in the brain. For example, humans, but not monkeys, can speak and use grammar, can reflect on their own mental states and those of others and can achieve an explicit understanding of causes in the physical and mental world.

## Differences between the human and monkey brain

We already know some of the specializations of the human brain that make this possible. They are summarized in a recent book by the author [16^••^]. First, the human brain is 4.8 times the size for a hypothetical monkey of the same body weight [17]. To put this into perspective, if one relates the size of the brain to the size of the medulla in the brain stem, the gap between the human brain and that of the macaque monkey is twice as large as the gap between the monkey and a small insectivore such as a shrew [16^••^].

But the human brain is not just a scaled up version of the monkey brain [18^•^]. The proportions of the human brain are not those that would be predicted by a plot of the changes in proportions in other primates as brain size increases. For example, the neocortex is 35% larger than predicted for a primate with as large a brain [19]. The prefrontal cortex, defined as the granular frontal cortex, forms 28.5% of the neocortex in the human brain but only 11.3% in the macaque brain [20^••^,21]. When related to the brain as a whole, the frontal polar cortex, area 10, is proportionately twice as large in the human brain as in that of the chimpanzee [22]. It is not even clear whether area 10 in the gibbon is homologous with the dorsal area 10 in the human brain [22], a fortiori for the macaque monkey.

There are two consequences of an increase in size. The first is that there is an increase in the number of specialized subregions, for example in the visual areas and in parietal cortex [23,24^••^]. This follows the general trend within mammals that there are more specialized sensory areas with increasing size of neocortex, perhaps because of the necessity to decrease the length of connections between similar inputs [25]. There is also a principle within sensory and motor systems that the amount of tissue devoted to a particular body part relates to the sophistication of the analysis or control rather than the size of that part. The amount of information received by the eye of a monkey and a human does not greatly differ, and yet the inferior temporal cortex is 12 times larger in the human brain [26].

The second is that there are consequential changes in the microstructure. The maximum spine density of layer III pyramidal neurones in the prefrontal cortex is 70% greater in the human than in the macaque brain [20^••^]. It is true that the value for the human brain is what would be predicted for a primate with a granular frontal cortex that was as large [20^••^]. But not all the differences in microstructure are the result of differences in size. For example, Buxhoeveden *et al*. [27^••^] measured the width of the mini-columns and the distance between columns in area Tpt within Wernicke's area. In the human brain the column width is 14–17% larger on the left than the right, whereas there is no such asymmetry in the columns of the macaque brain. There are also more magnopyramidal cells in the left rather than right superior temporal cortex in the human brain [28], and in the left rather than the right Broca's area [29].

There are other aspects of the microstructure of the human brain that cannot be accounted for by differences in size. Two of these have been discussed in relation to the human ability to reflect on one's own thoughts and those of others. The first is that the paracingulate area 32, which is activated when participants reflect on mental states [30], probably has no homologue in the macaque brain [31]. The second is that there are ‘spindle cells’ or ‘von Economo neurones’ in the anterior cingulate cortex and anterior insula of the human but not macaque brain [32^•^].

This is not to claim that these are the only possible differences between the human and macaque brain. But those mentioned above are enough to challenge the macaque monkey model.

## The usefulness of the macaque monkey model

So can the macaque monkey model survive this challenge? It is illuminating to start with the even more problematic task of generalizing from the rat brain to the human brain. The neocortex, with its white matter, forms just 28% of the brain in the rat, compared with 72% in the macaque monkey. This might make us very wary of adopting a rat model of the human brain. Yet, the hippocampus is well developed in the rat brain, and it was the discovery that the rat hippocampus is specialized for spatial mapping [33] that led to the explanation for the role of the hippocampus in episodic memory in the human brain. There is activation in the human hippocampus when subjects negotiate their way through space [34], but also specifically in the left hippocampus when they recall episodes in their life [35]. The reason is probably that personal episodes are remembered in their spatial, as well as their temporal context. Whether animals have the same experience of recollection when they remember is unclear, but what is clear is that the human brain has adapted mechanisms that exist in the rat brain.

The reason is that evolution is opportunistic, as we know not only from comparative anatomy and embryology but also from recent comparisons of the coding sequences of the DNA in different animals. Evolution is a historical process. It works in two ways. Where something works it retains it; where novel changes are required, they are typically made by adapting what was there in the first place. It is for this reason that the macaque monkey model can remain productive even in cases where humans have cognitive abilities that have not developed in other animals.

Take language, for example. It is controversial to what extent chimpanzees can be taught the elements of language. In particular, it is not clear that when chimpanzees are taught to use symbols, they appreciate that the aim is to influence the mental states of others, in the way that is characteristic of humans. But that does not mean that the macaque monkey model has nothing to contribute to the understanding of language. An essential characteristic of human language is that there is an arbitrary link between a word and its referent. That link must be learned because it is different in each particular language. There are pair coding neurones in the temporal lobe of macaque monkeys that can code for the learned association between two stimuli [36]. This is not to argue that all there is to reference is association, but a mechanism for association is still a necessity to allow the retrieval of a word. Of course, the associations in language can be cross-modal, as in the link between spoken words and their referents, but there are also cells in the monkey brain that can code for cross-modal associations [37]. When Japanese subjects learn the association between unfamiliar Korean or Thai ideograms and phonemes, there is activation in the superior temporal sulcus, just as for intelligible speech [38].

Humans differ from monkeys not only in being capable of language but also in being able to reflect on the thoughts of others. When they do so there is activation in the paracingulate cortex, area 32 [30]. One's first thought is that studying the monkey brain will tell us nothing about the mechanisms. But it is clear that the anterior cingulate cortex is involved in social evaluation even in other animals. Rats with lesions there show a decrease in social behaviour [39]. It is easier to analyze the reasons with monkeys, and Rudebeck *et al*. [40^•^] showed that macaque monkeys with cingulate lesions show a marked reduction in their interest in other individuals. The authors suggest that the anterior cingulate cortex is important for social valuation. This is not to claim that the monkeys could infer the mental states of other monkeys, but that they were less interested in the social signals made by other monkeys. In understanding the role of the paracingulate cortex in theory of mind, it may well be fruitful to understand the mechanisms for social valuation in monkeys.

## Conclusion

The previous section has deliberately taken difficult cases. There are, of course, many respects in which human abilities can be found in monkeys. In these cases one can give the same tasks to monkeys and human subjects. Examples are visual conditional tasks [41,42^•^], spatial working memory tasks [43,44], oddity tasks [45,46] or visual matching and non-matching rules [47,48].

Given uncertainty as to whether data on macaques can be generalized to humans, the strategy should be to follow up imaging experiments in human subjects by giving the same tasks to macaque monkeys in the fMRI scanner [24^••^,49]. This enables us both to interpret the fMRI signal in relation to electrophysiological signals and to visualize the similarities and differences between activations in monkeys and humans. Where there are similarities, one may then be justified in using data from the recording of single units and field potentials in monkeys so as to suggest mechanisms in the human brain. Wherever possible, one should try to confirm the results by direct recording from cells in patients, as has been done for recordings in the anterior cingulate cortex [50,51^••^].

We should also use the fact that we can intervene in monkey brains to check findings on the human brain. If A causes B, then removing A should prevent B. To give one example of our own, measures of effective connectivity in our imaging data suggested changes in the interactions between areas during learning [41]; and so we checked whether the changes in covariance were causal by studying the effect of disconnecting the relevant areas in macaque monkeys [42^•^]. The technique of disconnecting areas by crossed asymmetrical lesions provides an essential analytic tool for studying interactions that is not available for human subjects [52]. There will always be interventions that are needed if we are to study mechanisms but which we cannot make in the human brain: for example, selective depletion of different transmitters can reveal their contribution to the workings of specific areas in monkeys [53]. Finally, wherever possible we should directly compare the anatomical connections in the human and monkey brain [54^••^,55].

But why bother about monkeys at all? The reason is that recordings from electrodes in the human brain are always going to be restricted for ethical and practical reasons. For example, recordings can be taken for short periods during surgery for temporal lobe epilepsy [56] and for longer periods with depth electrodes implanted so as to detect the source of the seizure onset [57]. In these cases the aim of the recordings that are made for experimental purposes is not the clinical well-being of the patient, and there will always be strict limits to this type of research. However, recordings can also be taken so as to guide prostheses and here there is a clear clinical justification. Nonetheless, the basic work on decoding the activity of populations of cortical cells has first to be pioneered on macaque monkeys [58].

The message is clear. Work on monkeys is essential for understanding the mechanisms of the brain. But whenever possible, one should test whether the results can be generalized to the human brain. There is nothing outlandish about this message. After all, Kandel and co-workers [59] had to check whether the molecular mechanisms that they had established for learning in the sea slug (*Aplysia*) were also involved in memory in a mammal, such as the mouse. The gap between macaque monkeys and humans is of the same order, and we should follow their example.

## References and recommended reading

Papers of particular interest, published within the period of review, have been highlighted as:

• of special interest
•• of outstanding interest
